# 

**Published:** 1889-07

**Authors:** 


### BOOKS AND PAMPHLETS RECEIVED.

A System of Obstetrics. By American authors. Edited by B. C. Hirst, M. D. Volume II. Illustrated with 221 engravings on wood. Imperial 8vo; pp. xi., 854. Philadelphia: Lea Brothers & Co. 1889.

Synopsis of Human Anatomy. Physicians' and Students' Ready Reference series. By James K. Young, M. D. Illustrated. 12mo; pp. ix., 393. Philadelphia and London: F. A. Davis. 1889. Price, $1.40 net.

Lectures on the Errors of Refraction and their Correction with Glasses. Delivered at the New York Post-Graduate School, with illustrative cases from practice, both private and clinical. By Francis Valk, M. D., Lecturer on the Diseases of the Eye, New York Post-Graduate Medical School, etc. New York and London: G. P. Putnam's Sons. 1889.

Lectures on Bright's Disease. By Robert Saundby, M. D., Edinburgh, Fellow of the Royal College of Physicians, London; Emeritus Senior President of the Royal Medical Society, etc. With fifty illustrations. E. B. Treat, 779 New York. 1889.

Transactions of the Medical Society of the State of New York, for the year 1889. Published by the Society. William J. Dornan, printer, Philadelphia.

Transactions of the Washington Obstetrical and Gynecological Society. Vol. I. October 2, 1885, to May 20, 1887.

The Physiology of the Domestic Animals. A Text-Book for Veterinary and Medical Students and Practitioners. By Robert Meade Smith, A. M., M. D. Professor of Comparative Physiology in the University of Pennsylvania; Fellow of the College of Physicians and Academy of the Natural Sciences, Philadelphia, etc. With over 400 illustrations. F. A. Davis: New York and London. 1889.

Nouveau Traitment Chirurgical des Malades Inflammatoris des Reins et des Uretères Chez le Femme. Par Sherwood Dunn, Ph. D., Docteur en Médicine de la Facultié de Bellevue Hospital, N. Y.; Docteur en Médicine de la Facultié de Paris, etc. Avec figures daus le texte, une planche hors texte. Paris: Octaivé Doin, Editeur, 8 Place de l' Odeon. 1889.

A Clinical Study on Alopecia Areata and its Treatment. By L. Duncan Bulkley, A. M., M. D., New York. Reprint from the *Medical Record*, March 2, 1889. New York.

On the Value of Frequently Repeated Doses of Arsenic in the Treatment of Bilious Diseases of the Skin, especially in Children. By L. Duncan Bulkley, A. M., M. D. Reprint from the *New York Medical Journal* for April 13, 1889. New York.

On Unusual Methods of Acquiring Syphilis, with Reports of Cases. By L. Duncan Bulkley, A. M., M. D. From the *Medical News*, March 2 and 9, 1889.

A Résumé of Experience at the Aural Clinic of Prof. Hermann Schwartze, in Halle, Germany. By Charles H. May, M. D., Visiting Ophthalmic and Aural Surgeon, Randall's Island Hospitals; Assistant Surgeon New York Ophthalmic and Aural Institute, etc. Reprint from the *New York Medical Journal*, May 25, 1889.

Is More Conservatism Desirable in the Treatment of the Joint Diseases of Children? By A. B. Judson, M. D., Orthopedic Surgeon to the Out-Patient Department of the New York Hospital. Reprint from the *Medical Record*, May 18, 1889.

Report on the Treatment of Fourteen Cases of Disease of the Spinal Cord, by the Method of Suspension. By David D. Stewart, M. D., Lecturer on the Diseases of the Spinal Cord and Chief of the Medical Clinic in the Jefferson Medical College; Physician to St. Christopher's Hospital for Children. Reprint from the *Medical News*.

Ten Consecutive Cases of Abdominal Section for the Removal of the Uterine Appendages: For the Relief of Pelvic Pain and the Recurrent Attacks of Pelvic

Inflammation. A Report to the Ohio State Medical Society, at Youngstown, O., May 22, 23 and 24, 1889. By Rufus B. Hall, M. D., Professor Gynecology, Cincinnati Polyclinic; Clinical Lecturer on Gynecology, Miami Medical College; Fellow British Gynecological Society of London, etc. Cincinnati.

Scarlatinous Otitis. By Charles H. May, M. D., Visiting Ophthalmic Surgeon, Randall's Island Hospitals, New York, etc. Reprint from the *American Journal of Obstetrics* for April, 1889.

The Rational Method of Preventing Yellow Fever on the South Atlantic Coast. By J. C. LeHardy, M. D., Savannah, Ga. Read before the Medical Association of Georgia, at Macon, Ga., April 18, 1889.

Desmoid (Fibroid) Tumor of the Abdominal Walls. By Edward J. Ill, M. D, Reprint from the Transactions American Association of Obstetricians and Gynecologists, 1888.

De La Lobéline dans la Thérapeutique de L'Asthme. Mémoire présenté au I er Congrès Brèsilien de Médicine et Chirurgie, et Lu, Devant le Même Congrès À la Séance du 15 Septembre, 1888. Par le Dr. Sil Nunes. Rio Janeiro, 1889.

The Radical Cure of Hernia. By Thomas W. Kay, M. D., Scranton, Pa. Reprint from the *Maryland Medical Journal*, March 3, 1888.

Bulletin of the Agricultural Experimental Station. Cornell University. VI. June, 1889.

Nineteenth Annual Announcement of the Cincinnati College of Pharmacy, University of Cincinnati, Session of 1889-90.

Catalogue of the Albany Medical College, Medical Department of Union University. Fifty-eighth Session, 1888–89. And Announcement for Session 1889–90. Albany, N. Y.

Buffalo Law School. Announcement and Register of the Law Department Niagara University. 1889–90.

Programme of the Academic Department, University of Cincinnati. Seventeenth Year, 1889–90, with the Catalogue of Officers and Students for the Year 1888–89.

Merck's Bulletin, May, 1889. New York, London and Darmstadt.

Proceedings and Addresses at a Sanitary Convention held at Hastings, Mich., December 3 and 4, 1888, under the directions of a Committee of the State Board of Health and a Committee of Citizens of Hastings. Supplement to the Report of the Michigan State Board of Health for the Year 1889. Lansing: 1889.

American Public Health Association. Preliminary Announcement of the Seventh Annual Meeting to be held at Brooklyn, N. Y., October 22, 23, 24, 25, 1889.

Monthly Bulletin of the New York State Board of Health, April, 1889.

Health in Michigan, May, 1889.

Tennessee State Board of Health Bulletin. Volume IV., No. 11, June, 1888.

Report of Deaths and Contagious Diseases for the Month of May, 1889, Newport, R. I.

Weekly Abstract of Sanitary Reports Nos. 21, 22, 23, 24, 25, 26.

Wood's Medical and Surgical Monographs. Vol. II., No. 3. June, 1889. General Orthopedics, including Surgical Operations. By Dr. August Schreiber.

Immunity Through Leucomaines. By Eusebio Güell Bacigalupi. Translated from the second French edition by R. F. Rafael, M. D. New York: J. H. Vail & Co. 1889.

Transactions of the New York State Medical Association. Vol. V. 1889. New York: J. H. Vail & Co. 1889.

---

### *PERSONAL.*

Dr. C. C. Frederick, of this city, has been appointed a member of the Board of Examiners in Midwifery, for Erie county, vice Dr. J. W. Keene, removed to California. This will be recognized as a most excellent appointment, as Dr. Frederick is thoroughly qualified for the place.

---

### *LITERARY NOTES.*

Journalistic.—Dr. Hobart A. Hare, one of the editors of the *University Medical Magazine*, will, after October 1, 1889, assume editorial charge of the *Medical News*. Dr. Hare is well-known as a writer of vigor, and is an able executive officer. The *News* is to be congratulated.

---

The Times and Register has absorbed the *Polyclinic*. Four journals have now been consolidated under the editorial management of Dr. William F. Waugh, and published under the auspices of the American Medical Press Association. Next!

---

The American Journal of Obstetrics, for July, will publish a complete abstract of the proceedings of the Section of Obstretrics and Diseases of Women, of the American Medical Association, at the late meeting in Newport, R. I.

---

The Cosmopolitan, for July, is a most interesting number. Among its articles may be mentioned: "The Clubs of Chicago;" "The Murder of Philip Spencer;" "The American Bonapartes;" "The Eiffel Tower;" and "Six Feet of Romance." These and others of absorbing interest, handsomely illustrated, make it an exceedingly attractive number. It is the cheapest and best of magazines. Try it.

